# “I Don’t Trust AI”: A Generic Qualitative Analysis of College-Aged Mental Health Clients’ Perceptions of Artificial Intelligence Used in Mental Health Counseling

**DOI:** 10.3390/bs16050754

**Published:** 2026-05-12

**Authors:** Daniel Bates, Carly Antor, Paige Kammeyer, William W. Lorey, Timothy J. Hakenewerth

**Affiliations:** 1 Psychology and Counseling Department, Truman State University, Kirksville, MO 63501, USA; cmr3457@truman.edu (C.A.); ro85483@truman.edu (P.K.); mdpi@lorey.co (W.W.L.); 2 Counseling and Social Work, University of Illinois Springfield, Springfield, IL 62703, USA; tim.hakenewerth@gmail.com

**Keywords:** artificial intelligence, counseling, client perspectives, mental health technology, qualitative research

## Abstract

This qualitative study examined college-aged client perceptions of artificial intelligence (AI) in counseling services. AI technologies are beginning to appear in mental health treatment; it is important to understand client voices and perceptions. A generic qualitative descriptive design was used. Fourteen participants with recent counseling experience were recruited through purposive sampling from university channels. Data was collected via open-ended survey questions and analyzed using a six-phase reflexive thematic analysis. Trustworthiness was established through multiple strategies including peer debriefing, audit trails, reflexivity practices, and prolonged engagement with data. Six major themes emerged: (1) conditional acceptance of AI for non-clinical tasks, (2) concerns about data security and privacy, (3) valuing the human core of counseling, (4) preference for human judgment in crisis situations, (5) expectation of informed consent and transparency, and (6) cautious optimism contingent on evidence and safeguards. Findings suggest that AI implementation in counseling should follow an adjunctive rather than replacement model, with careful attention to maintaining therapeutic alliance and protecting client privacy. Implications for counseling practice, training, and policy development are discussed.

## 1. Introduction

Counseling practices, agencies, and organizations are starting to include artificial intelligence (AI) tools in their treatment delivery. The adoption of large language models (LLMs), a class of AI, and AI-enabled mental health tools has rapidly accelerated; however the research addressing the ramifications for counseling from clients’ perspectives have largely been unexplored, with existing studies examining medical patient attitudes (Baldus et al., 2026), chatbot user experiences (Chaudhry & Debi, 2024), engagement and effectiveness reviews (Limpanopparat et al., 2024), qualitative LLM-use narratives (Luo et al., 2025), and population-level prevalence and usefulness (McBain et al., 2025). College students represent a particularly relevant population for examining AI perceptions in counseling, as Generation Z members have been characterized as “digital natives” who have little to no memory of life without smartphones or internet access (Prensky, 2001). Contractor and Reyes (2025) found that over 80% of college students reported using AI for academic purposes such as getting feedback and generating essays. This suggests that college students possess a familiarity with AI and its capabilities. Despite the prevalence of AI in higher education and its introduction in mental health settings, the implications for AI’s use in counseling and counselor education have been largely unexplored (Fulmer, 2019; Wajid et al., 2025).

The counseling profession has historically adopted technological innovations, starting with telephone counseling to modern telehealth counseling (Freske & Malczyk, 2021; Lustig, 2012; Robertson, 2020; Snoswell et al., 2022). It seems adolescents and young adults in the United States are turning to generative AI for mental health support. In a nationally weighted survey of 1058 adolescents and young adults (aged 12 to 21 years), 13.1% reported using generative AI for mental health advice, rising to 22.2% among 18–21-year-olds. Most users sought advice from AI at least monthly (65.5%) and rated the advice as somewhat or very helpful (92.7%) (McBain et al., 2025). However, what AI represents is a fundamentally different type of technology, one that introduces an artificial agent that is doing the helping rather than the technology assisting a person doing the helping (Fulmer, 2019). The presence of an AI agent in mental healthcare that has the capacity to listen (e.g., ambient AI), write soap notes, generate treatment plans and interpret assessments raises some serious ethical concerns about client consent, clinical outcomes, and confidentiality. Yet, as AI expansion continues to accelerate across healthcare settings, we argue that client voices must be central in decision-making regarding the implementation of AI in mental healthcare. Therefore, the purpose of this study was to examine college-aged mental health client perceptions of AI, specifically LLM-based tools, within counseling contexts using systematic qualitative inquiry.

### 1.1. Conceptual Framework

#### 1.1.1. AI Applications in Mental Health Treatment

Early AI applications in mental healthcare consisted primarily of rule-based expert systems (where applications followed a pre-planned “if–then” set of guidelines rather than learning from data) with limited capabilities (Luxton, 2014). Advances in machine learning and natural language processing (NLP) have expanded AI with LLMs now capable of generating human-like answers and engaging in lengthy therapeutic-style conversations (Song et al., 2024; Siddals et al., 2024; Laestadius et al., 2024). Current applications include chatbots delivering CBT techniques (e.g., Woebot (Fitzpatrick et al., 2017), Wysa (Inkster et al., 2018), Replika (Laestadius et al., 2024), SimSensei (Rizzo et al., 2016)), emotional support agents (e.g., Tess (Fulmer et al., 2018)), diagnostic assistants (Cho et al., 2019), and linguistic analysis tools for detecting mental health concerns (Calvo et al., 2017), with varying complexity and human monitoring.

Research demonstrates that AI-based interventions can positively impact mental health outcomes. Randomized controlled trials examining Woebot found significant reductions in depressive symptoms (Fitzpatrick et al., 2017), while chatbots like Tess reduced depression and anxiety symptoms in university students (Fulmer et al., 2018). Wysa users reported feeling comforted and supported through CBT and mindfulness-based prompts (Inkster et al., 2018). A systematic review indicated generally positive attitudes toward mental health chatbots, though engagement varied based on perceived helpfulness, usability, and privacy considerations (Abd-Alrazaq et al., 2019). However, most extant studies have examined standalone AI interventions (e.g., outcomes of users interacting with a CBT chatbot) rather than focusing on client’s broader perceptions of AI integration within established therapeutic care.

#### 1.1.2. Risks and Ethical Considerations

Studies have indicated potential risks, particularly with emotionally immersive chatbots. Research on Replika, for example, found that intimate conversations were associated with an emotional overreliance on the AI, and as conversations progressed, AI’s limitations became evident to the users resulting in feelings of disorientation (Laestadius et al., 2024). The “uncanny valley” discomfort, understood as a psychological uneasiness with the almost-but-not-quite human nature of AI (Mori et al., 2012), has been reported among users using different AI chatbots (Ciechanowski et al., 2019). Other ethical concerns include confidentiality, informed consent, professional boundaries, competence, transparency, cultural sensitivity, and cybersecurity (Fulmer et al., 2020; Luxton, 2014). Data privacy is particularly salient given the sensitivity of therapeutic content (Nosrati et al., 2020). The therapeutic alliance, central to counseling, may be undermined by excessive AI reliance (Torous & Roberts, 2017).

#### 1.1.3. Gaps in Current Literature

AI is rapidly expanding across multiple healthcare settings and systems with the AI healthcare market projected to grow from $11.2 billion in 2023 to $427.5 billion by 2032 (Faiyazuddin et al., 2025). AI is starting to be used for diagnostics, treatment planning, and virtual care in healthcare (Bajwa et al., 2021; Olawade et al., 2024). However, limited research explores client perceptions of AI being used within counseling contexts (e.g., Baldus et al., 2026; Chaudhry & Debi, 2024; Limpanopparat et al., 2024; Luo et al., 2025; McBain et al., 2025). Most studies center on technical feasibility and clinical efficacy rather than client experiences, preferences, and concerns. This gap is significant because mental health agencies and organizations are deploying AI tools into their treatment models. The organizational benefits of AI need to be weighed against the interests and concerns of client welfare (Kretzschmar et al., 2019; Luxton, 2014). The purpose of this study was to understand college-aged counseling clients’ perceptions of AI integration in counseling services via exploratory qualitative inquiry.

## 2. Methodology

### 2.1. Philosophical Framework and Research Paradigm

This study was guided by a constructivist–interpretivist paradigm, which views knowledge as socially constructed and shaped by individual experiences and the meanings they assign to them (Lincoln & Guba, 1985). From this perspective, there is no single objective reality regarding the role of AI in counseling; rather, understandings of AI emerge through personal, relational, and contextual influences.

A constructivist stance was particularly appropriate given the quickly evolving nature of AI technology in mental health, where clients’ perceptions are still evolving. Our aim was to describe how clients currently make sense of AI’s presence and potential role in counseling, producing practice-relevant insights grounded in participant meaning-making.

We used a generic qualitative descriptive design (Sandelowski, 2000, 2010), an approach well suited for studies seeking to provide clear, low-inference descriptions of participants’ perspectives in their own words. Generic qualitative description allows researchers to stay close to participants’ language and meanings without imposing the procedural or theoretical commitments of more bounded qualitative traditions (Colorafi & Evans, 2016).

This design was intentionally selected for its pragmatic utility. Given the pace at which AI-related technologies are entering mental health practice and discourse, there is a need for timely, accessible accounts of client perspectives that can inform ethical decision-making, policy development, and clinical conversations. Our approach supports this goal through prioritizing participants’ own interpretations and emphasizing findings that are readily interpretable by counselors, supervisors, clinical directors, and other decision-makers who are considering how to ethically integrate new technologies into practice. Consistently with Sandelowski’s (2000) framework, this study aims to offer a coherent, analytically grounded description of how clients perceive AI in counseling contexts.

### 2.2. Participants

Consistently with best practices in qualitative counseling research (Hays & Wood, 2011; Hays & McKibben, 2021), we selected participants based on their ability to provide information-rich accounts relevant to the research questions. We used purposive criterion sampling (Patton, 2015), to recruit individuals with recent counseling experience and the ability to reflect meaningfully on AI integration in mental health. Sample size was guided by the concept of information power (Malterud et al., 2016), which suggests that smaller samples are appropriate when study aims are narrow, participants have direct experiences with the phenomenon, and analytic procedures are systematic. The final sample of 14 participants was deemed adequate given: (a) the narrow study aim, (b) participants’ direct counseling experience, (c) rich, open-ended responses, and (d) an iterative, team-based thematic analysis process.

Inclusion criteria required participants to be at least 18 years old, to have received counseling services within the past five years, to be fluent in English, and to be willing to share perspectives on AI in counseling. We limited inclusion to individuals who had received counseling within the past five years so that participant perspectives reflected recent changes in therapeutic practices and the latest developments in technology. Exclusion criteria included inability to provide consent, lack of recent counseling experience, or inability to complete the English-language survey.

Fourteen participants were recruited from a Midwestern liberal arts university through email notifications to student addresses. This approach provided access to individuals likely to have counseling experience. According to data collected in fall of 2025 via the National College Health Assessment, 26.0% of college students reported receiving counseling in the past 12 months (American College Health Association, 2026). These rates notably exceed data from the Centers for Disease Control and Prevention (2025), which indicates that approximately 14% of U.S. adults (all ages) received counseling or therapy from a mental health professional in the past 12 months, suggesting that college students access counseling services at substantially higher rates than the general adult population.

Our sample included eight women and six men, all aged 18–23. Eight were currently engaged in counseling, and six reported recent past experience. Most participants identified as single, White, lower socioeconomic status, Midwest residents, and Christian. Political affiliations included six Democrats, one Republican, three Independents, and four who declined to specify. Sexual orientations included eight heterosexual, three homosexual, and three bisexual participants. Although the sample was relatively homogeneous in age, education, and region, this enhanced *information power* by reducing variation unrelated to the research questions, while still providing sufficient diversity in counseling experiences.

### 2.3. Data Collection

Data were collected through an online survey administered via Google Forms. The survey included demographic questions and a series of open-ended prompts designed to elicit paragraph-length responses about participants’ perceptions of AI in counseling (see Supplementary Materials). These prompts allowed participants to respond in their own words, producing rich accounts that captured complexity and unanticipated themes (Creswell & Poth, 2018; Patton, 2015). Questions were developed from the study objectives and a review of the literature on AI in mental health, and they invited participants to share attitudes, perceived benefits and risks, conditions for acceptability, and preferences for implementation.

Following Institutional Review Board approval, recruitment emails with secure survey links were distributed to potential participants. Before beginning, participants reviewed informed consent materials describing the study’s purpose, procedures, potential risks and benefits, confidentiality protections, and voluntary participation rights. After providing electronic consent, participants completed the survey, which required approximately 15–20 min. To encourage participation, respondents could optionally enter a raffle for a $20 Amazon gift card, in which email addresses were collected separately to preserve anonymity.

### 2.4. Data Analysis

#### 2.4.1. Analytical Framework

We analyzed the data using Braun and Clarke’s (2006, 2019) six-phase reflexive thematic analysis, which is compatible with a constructivist, generic qualitative descriptive design. This approach provided a systematic yet flexible framework, allowing us to identify patterns across the data while staying firmly rooted in participants expressed meanings (Braun & Clarke, 2019). The six phases included: (1) familiarization through repeated reading and note-taking; (2) initial coding to capture explicit participant meanings; (3) clustering codes into preliminary themes; (4) reviewing and refining themes; (5) defining and naming themes with clear boundaries; and (6) producing the final analysis with illustrative excerpts.

#### 2.4.2. Analytical Process

Analysis was conducted collaboratively by the research team. Team members independently coded the dataset line by line, then met to compare interpretations, refine codes, and develop preliminary themes. Through iterative process of discussions, themes were reviewed, clarified, and refined until consensus was achieved, with all final themes grounded in multiple participant excerpts.

As a supplementary comparison strategy, an anonymized version of the dataset was also reviewed using ChatGPT (v. 3.5), consistently with prior work demonstrating AI’s utility as a secondary comparison tool in qualitative analysis (Christou, 2023; Hamilton et al., 2023; Marshall & Naff, 2024; Morgan, 2023; Wachinger et al., 2024). Specifically, the anonymized participant responses were submitted to ChatGPT (v. 3.5) in a single session using the following standardized prompt: “Please read these participant responses and identify recurring topics, patterns, and themes across the data. List the major themes you observe and provide brief descriptions of each.” This prompt was administered three times across three separate sessions, and the outputs were recorded and compared for internal consistency. The AI-generated theme lists were then compared side by side with the themes independently developed by the human research team. Convergence was defined as AI-identified topics that substantially matched a human-coded theme in focus and participant evidence; divergence was defined as topics flagged by AI that did not correspond to a human-coded theme, or human-coded themes that were absent from AI output. Divergent areas were returned to the raw data for additional interpretive scrutiny. Importantly, AI-generated outputs were not treated as analytic findings and did not replace human coding or interpretation. Rather, they served as a reflexive comparison point to examine areas of convergence and divergence, and to prompt the research team to interrogate their own interpretive choices.

Where AI-generated observations aligned with the research team’s analysis, they provided additional confidence in pattern recognition. Where discrepancies emerged, we returned to the raw data to evaluate interpretive accuracy and meaning. We tracked our coding decisions and team reflections to support analytic transparency, making sure that the final themes remained consistent with participants’ voices instead of automated outputs.

#### 2.4.3. Trustworthiness and Rigor

Trustworthiness was addressed by using Lincoln and Guba’s (1985) criteria of credibility, transferability, dependability, and confirmability, supplemented by contemporary considerations for qualitative rigor and recent guidance on the use of ChatGPT in analysis (Christou, 2023).

Credibility was enhanced by prolonged and iterative engagement with the data over approximately 18 months, with regular team meetings to interrogate interpretations, and systemic comparison of independent coding. Disagreements were explored through dialogue, allowing themes to be refined through critical examination. Transferability was facilitated by providing detailed descriptions of the participants, setting, data collection procedures, and analytic process, allowing readers to assess the applicability of their findings to other contexts. Dependability was supported through the maintenance of an audit trail documenting coding decisions, theme development, and methodological refinements. Team-based analysis additionally reduced reliance on any single researcher’s interpretive lens. Additionally, the use of a methodological consultant (Hakenewerth) provided consistency and coherence with study design, analytic coherence, and qualitative counseling research best practices. Confirmability was addressed through reflexive documentation of analytic decisions and explicit attention to how interpretations were grounded in participant data, including the use of illustrative quotations for each theme.

### 2.5. Researcher Positionality and Reflexivity

As the primary researcher (Bates), I have 16 years of experience as a licensed professional clinical counselor and counselor educator with a particular interest in technology’s role in counseling. This background informed how I understood participant responses, while also raising the possibility that my professional lens and assumptions about therapeutic relationships could influence interpretation. The research team also included three counselor-in-training graduate students (Antor, Kammeyer, and Lorey), whose perspectives as emerging professionals offered relevant perspectives on current counseling practice and client needs. Additionally, our research team included a counselor educator and clinician who served as a methodological consultant (Hakenewerth) who was not directly involved with data collection and analysis. Our different professional stages created multiple viewpoints, but also called for careful attention to how our roles might shape analysis. Within the team, perspectives on AI varied: some members were enthusiastic about its potential and saw new developments as exciting, while others were more cautious or uneasy about where it was heading. We reflected on these differing attitudes and how they might influence interpretation, using them as opportunities to challenge assumptions and stay grounded in participant perspectives.

Given the inclusion of AI as a supplementary comparison tool, reflexivity also extended to how the research team conceptualized and interacted with AI itself in the research process. Team discussions explicitly examined the limits of AI-generated pattern recognition, the risk of privileging coherence over nuance, and the ethical implications of integrating AI into qualitative workflows. These reflections informed our decision to position AI as a secondary, confirmatory resource rather than an analytic authority.

To address these dynamics, we engaged in systematic reflexivity throughout the project. We documented possible biases, examined our assumptions about AI in counseling, and worked collaboratively to reach consensus on coding and theme development. Regular team debriefings helped us distinguish between participant-driven themes and researcher assumptions, making sure that findings reflected client perspectives rather than professional judgments about AI in counseling.

## 3. Findings

Analysis of participant responses revealed six interrelated themes capturing college-aged client perceptions of AI in counseling: (1) conditional acceptance of AI for non-clinical tasks, (2) concerns about data security and privacy, (3) valuing the human core of counseling, (4) preference for human judgment in crisis situations, (5) expectation of informed consent and transparency, and (6) cautious optimism contingent on evidence and safeguards. Across themes, participants described conditional openness, concerns and reservations, and wariness rather than uniform endorsement or rejection. To protect participant confidentiality, participants are identified using pseudonyms throughout the presentation of findings.

### 3.1. Conditional Acceptance of AI for Non-Clinical Tasks

Participants commonly expressed limited and conditional acceptance of AI applications in administrative and logistical functions, while maintaining firm boundaries against AI involvement in direct therapeutic interactions. AI was viewed as potentially appropriate for supportive tasks that could reduce counselor administrative burdens, such as scheduling, basic note-taking, and appointment reminders, particularly if such use reduced counselor workload and allowed more time for clinical care. Alex explained, “More administrative tasks such as scheduling, invoicing, etc.… I am completely in favor of,” reflecting a perspective shared by several participants. Similarly, Drew noted openness to “management tasks, scheduling reminders, etc. Basically anything that would ease their burden without feeding any patient information (outside of scheduling reminders) into an AI,” emphasizing that these uses were acceptable when they did not intrude into the therapeutic space.

However, acceptance was rarely unconditional. Even participants supportive of administrative uses expressed concerns when tasks intersected with clinical notes or records. Alex added, “tasks like note-taking and record-keeping I feel cautious about given the lack of privacy and data security Gen AI tools tend to carry with them.” These responses illustrate that openness to administrative AI was often bounded by concerns that reappeared in subsequent themes, particularly around privacy and data protection.

### 3.2. Concerns About Data Security and Privacy

Concerns about data security and privacy emerged as a central and persistent barrier to AI acceptance in mental health settings. Participants worried if AI could truly keep private counseling conversations safe. Many described AI as inherently vulnerable to hacking, unauthorized access, or the misuse of confidential data, possibly undermining the foundational expectation of confidentiality in counseling.

Several participants expressed discomfort with the idea of personal disclosures being stored or processed by AI systems. Emily stated, “I would not want AI to be involved when discussing my feelings. I would not want my personal experiences and emotions being documented into a database.” Others emphasized the relational dimension of confidentiality. Ava shared, “I just worry that details of my life that I share in confidentiality with my counselor won’t stay within the room and meeting if AI is also present.”

For some, the issue was tied to a broader lack of trust in institutions. Ethan bluntly stated, “I trust AI about as much as I trust the government.” In contrast, some participants described their concerns as being conditional or time limited. Alex noted, “I do not trust AI’s ability to handle sensitive data in its current state, although I could see this improving in the future.” Together, these responses highlight variation in how participants conceptualized risk, ranging from absolute rejection to cautious, future openness. Beyond questions of data security, participants also raised concerns about the relational and emotional implications of AI in counseling, examined in the next theme.

### 3.3. Valuing the Human Core of Counseling

Participants frequently emphasized their belief that counseling is a fundamentally human and relational experience, and many expressed concern that increased reliance on AI could erode or replace what they viewed as the essential human qualities of therapy. Their responses reflected a broader commitment to protecting the human core of counseling, by valuing what human counselors provide while remaining wary of how AI might reshape or diminish those qualities.

Across interviews, participants expressed doubts about AI’s capacity to provide emotional support and understanding necessary for effective therapeutic relationships. Several participants perceived AI’s use in counseling as detached and devoid of empathy. Madison shared, “To me, the process of AI use in counseling seems incredibly impersonal.” Others emphasized the irreplaceability of human therapeutic presence: “AI cannot lend a listening ear the same way a counselor could,” said Emma, while Morgan added, “It takes out the human understanding.”

Some participants expressed these views more forcefully. Alex stated, “AI should NEVER engage with clients. The intangible and immeasurable significance of a human connection in the therapy room is of the utmost importance.” Olivia framed her concerns more symbolically, observing, “The main difference that stands out to me is the removal of humans from something that I consider being a very human field.” Similarly, Emma reflected, “Traditional counseling gives you a sense of comfort in this rising digital age. AI-assisted [sic] lacks emotion, sympathy, and understanding,” while Jake noted, “It’s not a real person, so you’re not gonna see real empathy.”

For many participants, these relational concerns were intertwined with fears that AI could eventually displace or replace human counselors or the human elements of therapy. Rather than rejecting technology outright, they tended to draw a line around what they believed should remain distinctly human. As Riley explained, “I hope it is used to make the business run more efficiently, I fear it will replace the human connection with something artificial.” A smaller number of participants connected these concerns to the future of the profession itself. Hannah shared, “As a future potential counselor, I fear AI will take my job from me.” These responses illustrate that fears of replacement were not solely about employment but also about the potential loss of relational depth, confidentiality, and human judgment that participants associated with effective counseling—qualities they believed AI couldn’t replicate.

Overall, participants’ responses in this theme revealed a nuanced stance: while they could imagine limited, supportive uses of AI, they were deeply invested in preserving the human emotional presence, empathy, judgment, and connection they viewed as central to therapeutic healing. Participants connected these fears to broader anxieties about the dehumanization of healthcare and the erosion of authentic interpersonal connection. For them, replacing human therapists with AI was not just a change in service delivery, but a fundamental threat to the very nature of counseling. Their concerns about replacement were best understood not as blanket technophobia, but as an effort to protect what they believed makes counseling meaningful and effective.

### 3.4. Preference for Human Judgment in Crisis Situations

Participants voiced serious concerns about whether AI could ever respond appropriately in a mental health crisis. Many stressed that crisis intervention requires human judgment, emotional sensitivity, and the ability to adapt in the moment, qualities they did not believe AI currently possesses. While participants differed in their general openness to AI, there was near-universal agreement that crisis intervention should remain the responsibility of trained human professionals.

Several participants described fears that AI’s reliance on algorithmic protocols could be harmful in urgent situations. Emma cautioned, “In a crisis, such as someone contemplating suicide, AI’s lack of human ability could draw someone to continue taking their life. AI is good at following protocol, not adapting to the situation.” For others, the problem was broader skepticism about AI’s basic reliability. Chris remarked, “AI can’t even do a basic math problem, we are screwed if people start relying on it too heavy [sic]!”

When asked directly about AI’s role in crisis response, participants were emphatic in their rejection. Olivia captured this unequivocal attitude in her quote: “Not at all. That is something I would expect the human counselor to do.” These responses underscore participants’ belief that crisis situations demand immediate human presence, clinical judgment, and ethical responsibility, qualities they didn’t associate with AI systems.

### 3.5. Expectation of Informed Consent and Transparency

Participants consistently emphasized that they would want transparency and informed consent if AI was used in any part of their counseling. Disclosure about AI involvement was viewed not only as an ethical obligation but as a condition of trust and continued engagement in therapy. Madison explained, “I believe that persons undergoing counseling that uses AI assistance need to be aware that it is being used as well as if the information is stored and the potential risks that pose to them as individuals.” Emily similarly emphasized, “I would want the informed consent process to be made very clear.”

For some participants, knowing about AI use was a decisive factor in whether they would remain in counseling at all. Olivia captured this bluntly, saying, “If a counselor was using AI in or related to my sessions I would stop going to them.” These responses illustrate that disclosure was not merely informational, but central to participants’ sense of agency and choice within the counseling relationship and their participation.

### 3.6. Cautious Optimism Contingent on Evidence and Safeguards

Despite significant concerns, participants expressed some cautious optimism about AI’s future role in counseling. Approximately half of the participants described openness to AI in counseling if supported by strong research evidence, clear ethical safeguards, and firm limits on what AI should and should not do. The other half, by contrast, described feeling “fear” or being “afraid” when imagining AI becoming more common in mental health care. For those who leaned more open, evidence was the key factor. Alex explained, “A wide and deep literature supporting the use of AI in counseling would change my opinion slightly more positive [sic].” Emma echoed that point: “Yes, the more research that comes out, the more reliable it would become. With enough positive information, I would likely be more open to AI.”

A few participants even imagined ways AI could add unique value to counseling. Emily noted, “My hopes of the future of AI in counseling are that it will be able to help people evaluate their emotions in ways that a human counselor may not think of.” This kind of response reflected curiosity about how AI might complement, rather than replace, human therapeutic skills. Overall, participants’ cautious optimism revealed a mix of openness and skepticism: they recognized that AI is still evolving, but they were clear that safety, effectiveness, and ethics would need to be proven before they would accept greater use in counseling.

## 4. Discussion

This study explored college-aged client perceptions of AI in counseling using a generic qualitative descriptive approach. Overall, participants articulated complex, nuanced, and conditional views, neither wholly rejecting, nor enthusiastically endorsing the integration of AI into counseling practice. It is important to note at the outset that the findings presented here reflect the subjective perceptions and verbal reports of 14 college-aged clients drawn from a single Midwestern university. As such, these perceptions should be understood as providing insight into how participants in this specific context made sense of AI in counseling, they are not intended as evidence of AI’s actual capabilities, risks, or clinical adequacy, nor are they necessarily generalizable to broader populations. The caution expressed by participants in this study is notable given that our participants consisted of college students, a population often characterized as digital natives (Prensky, 2001) who demonstrate high rates of AI use for academic tasks (Contractor & Reyes, 2025) and time management (Klimova & Pikhart, 2025). Despite their technological fluency and familiarity with AI, a pattern emerged, across themes, of cautious conditional acceptance, characterized by clearly articulated boundaries regarding how, where, and under what conditions AI might be acceptable within counseling contexts. Palfrey and Gasser (2008) wrote “For… young people, new digital technologies, computers, and cell phones are primary mediators of human-to-human connections” (pp. 4–5). And we can see from our participants that they expressed a protectiveness of that “human-to-human connection” in the counseling context.

Participants, broadly speaking, supported AI in administrative or supportive roles where it could reduce counselor workload without disrupting the therapeutic relationship. At the same time, participants drew firm boundaries around AI’s involvement in emotionally sensitive interactions, clinical judgment, or crisis response. These boundaries were consistently tied to concerns about privacy, trust, emotional connection, and ethical responsibility. This conditional acceptance reflects prior research suggesting that AI is most acceptable to users when framed as an adjunct rather than a replacement for human care (Fulmer et al., 2020; Liu et al., 2022; Ma et al., 2024). The present findings contribute to this literature by surfacing strongly felt, client-defined limits within a sample of college-aged counseling clients, such as the rejection of unsupervised crisis response or autonomous clinical decision-making, that may warrant further investigation across broader populations.

This study’s findings converge with the prior literature. For example, prior research indicates that users perceive AI chatbots as helpful adjuncts versus replacements for human therapists. For instance, participants who knew they were interacting with AI rated human responses as more authentic and practical, viewing AI responses as helpful yet supplemental (Jain et al., 2024). Qualitative data from individuals of diverse backgrounds using AI for mental health care found that users create unique support roles for chatbots while maintaining the necessity of human therapeutic care for long-term treatment (Song et al., 2024). Another study similarly indicated higher acceptance for use of AI-based conversation agents for use of mental health support when overseen by human healthcare professionals, not autonomous (Lukas, 2025). Expert panels interviewed by Ma et al. (2024) reported that LLM tools are most acceptable when they create efficiencies in clinicians’ clerical load (such as drafting progress notes or sending reminders) rather than when they carry on therapeutic dialogue on their own.

Concerns regarding what participants perceived as AI’s limitations in emotional attunement and crisis response were particularly salient. Participants repeatedly emphasized that counseling is experienced as an inherently human endeavor, grounded in empathy, responsiveness, and relational presence. These perspectives echo longstanding counseling scholarship emphasizing the centrality of the therapeutic relationship (Rogers, 1957; Wampold, 2015). While some prior studies have documented positive client experiences with AI-based mental health tools (e.g., Fitzpatrick et al., 2017; Inkster et al., 2018), those studies often examined AI as a stand-alone intervention. In contrast, participants in the present study evaluated AI within the context of an ongoing therapeutic relationship, which may help explain their heightened concern about relational displacement and ethical risk.

Consistently with the prior literature (e.g., Fulmer et al., 2020; Nebeker et al., 2019; Nosrati et al., 2020), participants framed trust in AI not solely as a technical issue but as an ethical and relational one, with some expressing that the absence of transparent disclosure would be sufficient reason to discontinue counseling. These concerns mirror recent warnings regarding the lack of HIPAA-equivalent protections in many consumer AI tools (Abrams, 2025). Importantly, the participant-identified priorities of informed consent, transparency, and human oversight align closely with guidance published by major counseling professional organizations. The American Counseling Association (American Counseling Association, 2024), the American Mental Health Counselors Association (American Mental Health Counselors Association, 2024), and the National Board for Certified Counselors (National Board for Certified Counselors, 2024) have each issued position statements or ethical guidelines emphasizing that counselors must disclose AI use to clients, obtain informed consent, safeguard client data, and ensure that AI tools do not supplant the therapeutic relationship. That participants in the present study independently identified priorities that parallel professional guidance, without exposure to those frameworks, is noteworthy, though it should be interpreted cautiously given the small and demographically homogeneous sample. If replicated in broader populations, such alignment could inform future efforts to center client voices alongside organizational guidance in developing institutional AI governance frameworks.

Our sample consisted of tech-savvy Gen-Z participants who questioned AI’s ability to empathize and manage high-risk scenarios, echoing concerns in Malouin-Lachance et al. (2025). Participants’ skepticism was especially pronounced in relation to crisis situations, where human judgment, adaptability, and ethical accountability were viewed as non-negotiable. Participants emphasized the irreplaceable role of trained professionals in high-stakes scenarios. These findings support the work of Torous and Roberts (2017), who cautioned that while AI may offer efficiency and convenience, it may not adequately replicate human judgment in complex or high-stakes clinical contexts. Importantly, participant responses were not uniformly pessimistic. Approximately half of the participants voiced a conditional optimism regarding AI’s future role in counseling, particularly if supported by rigorous research, ethical safeguards, and clear limitations. This cautious openness parallels findings from Siddals et al. (2024), who reported perceived benefits of AI for emotional labeling and self-reflection, while highlighting that participants in the present study emphasized the continued necessity of human clinician presence. Taken together, these findings suggest that, at least among the participants in this study, openness to AI in counseling was not uniform but appeared contingent on evolving evidence, transparency, and relational integrity.

### 4.1. Implications

While exploratory, these findings point to issues that may be relevant for counseling services serving digital-native populations. While college-aged clients may be more comfortable with technology and feel less stigma around mental health help-seeking, findings suggest that, at least for this sample, technological and AI familiarity did not translate to blanket acceptance of AI in therapeutic settings. Rather, participants’ responses highlighted the importance of approaching AI integration through a client-centered and relationally grounded lens. This is consistent with other studies finding that college students who regularly use AI for academic support nonetheless report awareness of AI hallucinations and misinformation risks, with 51% indicating that such awareness discourages their use (Neves et al., 2025). These findings suggest that counselor training on ethical AI use, particularly in how to communicate about it and uphold informed consent, may warrant attention.

The persistence and intensity of participants’ data privacy concerns, regardless of their source, underscore the importance of clear communication about how AI intersects with existing systems of record-keeping and data protection, rather than assumptions about client understanding.

### 4.2. Practice Implications

For practitioners, these findings suggest the value of a gradual and transparent approach to AI integration in counseling. Beginning with low-risk administrative applications while maintaining strict separation from clinical interactions with the client, sharing results openly, and checking back with clients as the technology develops may align more closely with client expectations. Clear language laid out in the informed consent materials should explicitly address AI involvement and data protocols, with transparency requirements including disclosure of all AI applications and ongoing communication about changes. Consistently with participants’ strong preference for human involvement in crisis situations, practitioners may wish to ensure that crisis response protocols remain under human oversight, with AI systems designed to defer to trained professionals in high-risk situations.

### 4.3. Training Implications

Counselor training programs may benefit from incorporating foundational education on AI, including its capabilities, limitations, and ethical practice considerations. Training should emphasize ethical decision-making, preparing counselors to advocate for client preferences, and communication skills related to discussing AI use with clients. Given participants’ emphasis on relational depth, training should continue to reinforce the centrality of the therapeutic alliance even as technological tools become more prevalent.

### 4.4. Policy Implications

Participants’ concerns suggest that professional organizations may wish to develop or expand ethical guidelines for AI use in counseling, with attention to client autonomy, informed consent, and the protection of the therapeutic relationship. Given the sensitive nature of counseling content, privacy standards may need to address concerns beyond general healthcare regulations. The present findings underscore the potential value of evidence-based requirements for AI implementation that prioritize client safety and preferences, along with transparency, client choice, and ongoing assessment of client satisfaction.

### 4.5. Limitations

Several limitations should be considered when interpreting these findings. The sample was drawn from a single Midwestern university population, and consisted of primarily single young adults between the ages of 18 and 23. As such, the findings may not transfer to the perspectives of older adults, individuals from different cultural or regional backgrounds, or clients with more extensive counseling histories.

Additionally, participants’ baseline familiarity with AI and its current capabilities likely varied considerably, and the study did not assess AI literacy prior to data collection. Responses may therefore reflect differing levels of understanding or exposure. The use of an online survey format also limited opportunities for follow-up probing or clarification, which may have constrained the depth and nuance of responses compared to data collected through interview-based methods.

### 4.6. Recommendations for Future Research

Future research could build on these findings by assessing participants’ AI literacy prior to data collections to better contextualize their perceptions. Given that concerns expressed by participants appeared to stem, at least in part, from varying levels of familiarity with the underlying technology, such as fears that AI would indiscriminately “document into a database” or mishandle private information, gauging baseline familiarity with AI could help differentiate between concerns rooted in technological reality and those driven by lack of exposure.

Diverse population studies including varied age groups, geographical regions, cultural backgrounds, and counseling histories would build our understanding of how demographic factors influence AI acceptance and preferences. Cross-cultural research could explore variations in AI acceptance related to cultural values regarding technology, privacy, and therapeutic relationships.

Finally, longitudinal or experimental designs may be valuable in assessing whether attitudes shift over time with greater familiarity or exposure, in addition to how clinical outcomes are affected by the use of AI in counseling. Future research could examine how ongoing interaction with transparent, ethically designed AI tools may influence client trust, perceived usefulness, and therapeutic outcomes, especially as the technology continues its rapid pace of advancement. Such research would provide a more dynamic understanding of how client perceptions evolve in parallel with technological advancement.

## 5. Conclusions

As AI technologies continue to progress and become more integrated into mental health care, centering client voices remains an important consideration. Using a generic qualitative descriptive design, this study provides insight into how college-aged clients currently understand and evaluate the role of AI in counseling. Participants articulated clear boundaries around acceptable uses of AI, expressing support for administrative and adjunctive functions, while rejecting AI involvement in emotionally sensitive, crisis, or ethically complex aspects of care. Across responses, participants emphasized the importance of preserving the intrinsically human nature of counseling, grounded in empathy and responsiveness. While some participants expressed cautious optimism about AI’s future potential, they did so conditionally, emphasizing the need for rigorous research, ethical oversight, and clearly defined boundaries.

In sum, these findings suggest that the integration of AI into counseling would benefit from proceeding thoughtfully and incrementally, guided by client values and ethical priorities. Ultimately, the way we bring AI into counseling will affect not just how services run but also the trust at the heart of the therapeutic relationship. Continued dialogue among counselors, researchers, educators, and policymakers will be critical for ensuring that new technologies preserve client trust, autonomy, and safety and enhance, rather than erode, the relational heart of the profession.

## Data Availability

Dataset is available upon request.

## Supplementary Materials

The following supporting information can be downloaded at: https://www.mdpi.com/article/10.3390/bs16050754/s1.
