# Supplementary material for: “I Don’t Trust AI”: A Generic Qualitative Analysis of College-Aged Mental Health Clients’ Perceptions of Artificial Intelligence Used in Mental Health Counseling
Source: Behav Sci (Basel). 2026 May 12;16(5):754. doi: 10.3390/bs16050754 (PMC13203814; doi:10.3390/bs16050754)
Supplement: Supplementary file 1 [file behavsci-16-00754-s001.zip › behavsci-4233401-supplementary.pdf]

Supplementary Materials

Qualitative Essay Questions

What tasks are you comfortable with your counselor using AI to complete during a session? (e.g., taking notes, recording sessions, summarizing, management tasks like invoicing, scheduling reminders, crisis management, tracking progress, etc.)

How do you see AI-assisted counseling compared to traditional counseling sessions? What differences stand out to you the most?

How do you feel about the confidentiality and safety of your information when AI is involved in the process? Do you trust the AI's ability to handle sensitive information?

In what ways, if any, do you feel that AI affects your relationship with your counselor? Does it enhance or hinder the connection?

How much of an emphasis would you want to be placed on the informed consent process for the use of AI in your counseling services?

What ethical concerns do you have about the use of AI in counseling?

Do you think AI can be trusted to keep your personal information confidential?

How comfortable are you with the idea of AI analyzing your speech and emotions during therapy sessions?

If there were proven benefits to using AI in counseling, such as better understanding your emotional patterns, would this increase your comfort level?

What are your hopes and fears regarding the future of AI in counseling?
